# Impact of cost sharing on quality improvement and profits under uncertain demand: The case of a textile and garment supply chain

**DOI:** 10.1371/journal.pone.0304578

**Published:** 2024-05-31

**Authors:** Qigui Lang, Jianfeng Hu, Jinjin Liu

**Affiliations:** 1 Zhejiang Sci-Tech University, Hangzhou, China; 2 Business School, Yangzhou University, Yangzhou, China; Sichuan Agricultural University, CHINA

## Abstract

The study explores the strategic pricing and quality improvement decisions under uncertain demand in a three-layer textile and garment supply chain. According to whether the fabric manufacturer (FM) invests in quality or not and whether the garment manufacturer (GM) or garment retailer (GR) is willing to share the costs or not, five game models are constructed to investigate the impact of different members’ cost sharing on the optimal decisions and profits. By conducting a theoretical and numerical analysis, we find that: (1) The GM’s or GR’s cost sharing plays a positive effect on the quality improvement, as for whose cost sharing performs better in improving the quality depending on the proportion of cost sharing, and the quality improvement is highest with both members share the costs simultaneously. (2) The FM receives the highest profit when both members share the costs simultaneously, however, whose cost sharing is more profitable for the FM is also related to the proportion of cost sharing; in short, the FM always benefits from the cost sharing, no matter one member does this or two members do this. (3) The GM (GR) gains the highest profit when only the GR (GM) shares the costs, and the results indicate that if one member has shared the costs, whether the other member engaging in cost sharing could benefit the former depending on their proportions. Specifically, when the GM (GR) chooses to share the costs and the proportion is relatively low, the GR(GM) joining in cost sharing is beneficial to the former; otherwise, is harmful.

## 1 Introduction

With the improvement in economic and living standards, consumers pay greater attention to products’ quality in addition to prices and begin to concern products’ safety and environmental protection aspects. Quality improvement as a powerful competitive tool is now adopted by many enterprises for attracting quality-sensitive consumers. In the textile and garment industry, in particular, clothing products are directly in contact with a consumer’s skin and are generally worn for a long time. If the fabric quality of the clothing is poor, it may harm consumers or shorten the service life of the clothing itself, such as via shrinkage, fading of color, creases, and other phenomena.

In response to consumers’ increasing emphasis on quality, more and more enterprises invest in technology or raw materials to improve product quality. For example, Anta has launched a variety of environmentally friendly fabrics such as SORONA^®^, fluoride-free waterproof fabrics, and actively promotes the use of sustainable packaging to create sustainable sporting goods and minimize the use of harmful chemicals. It can be seen from the financial report that in 2022, Anta invested nearly 1.3 billion yuan in R&D costs, and applied for more than 3,000 patents, which is almost a fault level leading the Chinese industry.

In practice, the garment enterprise usually needs to invest a lot of money to improve the fabric, the intermediate production process, etc., to improve the quality of the final product. Some large enterprises can cover the entire investment themselves, while some small and medium-sized enterprises may not be able to afford such high R&D expenses, in which case other members of the supply chain may be required to share some of the costs. However, whether the other members are willing to share the costs is uncertain. Moreover, how different members’ cost sharing affects quality improvement and profits is unknown and needs to be explored. In this way, we understand the impact of cost sharing on quality improvement and find out the optimal strategy to guide the decisions of enterprises in practice. Hence, this is a problem worth studying and urgently needs to be solved.

Due to the dynamic market environment and evolving consumer preferences, a product’s demand is often uncertain, especially in terms of new and improved products. Given that the garment industry reflects the fashion industry, the market demand is usually unpredictable and prone to uncertainty due to the rapid changes in fashion trends. However, in the face of uncertain demand, companies may be more conservative in their decisions, especially when it comes to large investments. Hence, it is necessary to take the uncertain demand into account to discuss its effect on the production, pricing, and quality improvement decisions as well as the other members’ cost sharing.

Based on the above discussion, this work studies the pricing and quality improvement decisions in a textile and garment supply chain with three members: an upstream fabric manufacturer (FM), a midstream garment manufacturer (GM), and a downstream garment retailer (GR), where the FM could decide whether to invest in quality improvement or not with the GM or GR able to choose whether to share the corresponding costs or not. Then our paper aims to address the following questions:

(1) How do the three members make the pricing and quality improvement decisions with uncertain demand?
(2) How do the members’ different cost-sharing behaviors affect the pricing and quality improvement decisions as well as profits?
(3) Whose cost sharing is more beneficial to the quality improvement and the three members?

To address these problems, we establish five game models by considering the FM’s quality improvement choice and other members’ cost sharing behaviors. Besides, the uncertain demand is captured with two states of high demand and low demand, which is a simplification of reality but which is sufficient to prove the impact of uncertain demand. We derive the equilibrium solutions under different cases by maximizing the members’ expected profits using backward induction and then discuss these results by conducting a theoretic comparison and numerical analysis.

In contrast to the previous research, the main contributions of our paper are as follows. First, for the textile and garment supply chain, this paper constructs a variety of game models to study the pricing and quality improvement decisions of enterprises from the theoretical level, which fills the deficiency of the existing literature in the field of garment supply chain that only focuses on empirical research. Second, we consider the uncertainty of clothing products in the market, describe the uncertain demand, and discuss the FM’s quality improvement decision under this background, which expands the scope of research on quality improvement in literature and provides a theoretical reference for enterprises’ decision-making in practice. Third, this paper compares the impacts of different members’ cost sharing behaviors on quality improvements and profits, which provides management advice for cost sharing decisions in supply chains.

The structure of this paper is as follows. A comprehensive review of the literature related to our research is provided in Section 2. Section 3 presents the problem description and demand function. In Section 4, we provide model and theoretical analysis under various scenarios, including no quality improvement, quality improvement without cost sharing, and quality improvement with cost sharing. Subsequently, based on the equilibrium solutions, we conduct comparison analysis in Section 5 and numerical analysis in Section 6. Finally, Section 7 summarizes the findings and managerial insights and proposes future research directions.

## 2 Literature review

The literature about our work encompasses three main areas: (i) textile and garment supply chains, (ii) uncertain demand in supply chains, and (iii) quality improvement and cost sharing.

### 2.1 Textile and garment supply chains

Most of the literature on textile and garment supply chains is done from an empirical perspective and explores how to optimize operational performance [1, 2]. Ma et al. [3] proposed a new collaborative model to integrate common and crucial collaboration strategies in the demand-driven garment supply chain. Ahmed and MacCarthy [4] explored the applications of blockchain technology in the textile and apparel industries, and analyzed a prominent example of a blockchain-based traceability solution adopted by Lenzing Group, a world-leading fiber producer. There is a paucity of literature regarding the mathematical modeling of textile and garment supply chains [5]. For instance, Wang et al. [6] proposed a multicriteria decision-making model to study supplier selection and evaluation in garment supply chains. To address the similar supplier selection and evaluation, Karami et al. [7] employed a three-step integrated approach in garment supply chains. Wang [8] studied the impacts of advertising efforts and water-saving in apparel supply chains. Differing from this existing literature, our work studies pricing and quality improvement decisions in a three-layer textile and garment supply chain from the perspective of mathematical modeling.

### 2.2 Uncertain demand in supply chains

Early, Fernando and Awi [9] investigated the equilibrium behavior of decentralized supply chains with competing retailers under demand uncertainty. Next, Shi et al. [10] examined how power structure affects supply chain members’ performance with random demand, and they capture the uncertain demand using a random variable. Giri and Bardhan [11] studied the coordination problem in a two-echelon supply chain under uncertain demand. Then, Wang and Song [12] investigated under uncertain demand, the pricing policy in a dual-channel supply chain with green investment and sales effort. Similarly, under demand uncertainty, Xin et al. [13] focused on the problem of coordinating a two-echelon green supply chain with environmentally conscious consumers. Recently, Gao and Zhao [14] considered extended warranty service in a sustainable and environmentally responsible supply chain, where the uncertain demand is addressed by uncertainty theory.

Based on the above literature, this paper also studies the effects of uncertain demand on pricing decisions and supply chain members’ profits. As an extension, this paper explores (1) the impact of uncertain demand on quality improvement; (2) pricing and quality investment decisions in a three-tier textile and garment supply chain; (3) how different members’ cost-sharing behaviors affect product quality and the members’ profits. These are the differences from the previous literature.

### 2.3 Quality improvements and cost sharing

Our paper is closely connected to the literature on quality improvements, which has been a prevalent topic in supply chain management [15–18]. Yoo and Cheong [19] proposed several incentive mechanisms for collaborative product quality improvement. Chakraborty et al. [20] discussed two competing manufacturers’ quality improvements and proposed a cost-sharing mechanism. In a three-echelon supply chain, Zhu [21] examined quality control strategies under different channel structures (direct channel, retail channel, and mixed channel). Fu et al. [22] studied the supplier’s investment in the manufacturer’s quality improvements under different market power structures. In an e-commerce supply chain, Wu and Zheng [23] investigated the impacts of quality improvement and information acquisition. Recently, Xu et al. [24] focused on the trade-off between quality improvement and price-matching under a competitive environment with a dual-channel structure. Similarly, we also consider the improvements in quality, while the difference is that we study a three-layer textile and garment supply chain in an industry where consumers are particularly sensitive to product quality.

Another topic related to our paper is cost sharing problem in supply chains [25–27]. This body of literature can be divided into two categories: one is concerned with contract selection between revenue sharing and cost sharing in supply chains; the second is cost sharing under different supply chain backgrounds, including closed-loop supply chains, green supply chains, and dual-channel supply chains. For instance, Xie et al. [28] discussed revenue-sharing and cost-sharing contracts in a dual-channel closed-loop supply chain. Cai et al. [29] considered cost sharing under two warranty policies in supply chains. In a low-carbon service supply chain, He et al. [30] investigated two types of cost sharing contracts: sharing emission reduction costs or sharing service costs. Liu et al. [31], Chen et al. [32], and Gong et al. [33] both studied cost-sharing mechanism under different power structures, and Liu et al. [31] focused on improving product greenness while Chen et al. [32] considered remanufacturing process innovations. Much of the existing literature utilizes game theory to address the quality improvement [20, 22], cost sharing problems [34, 35]and investment decision [36, 37] in supply chains.

Similarly, our paper also considers one manufacturer’s quality improvement and one retailer’s cost sharing behavior, but the difference is that we choose a three-tier supply chain and discuss different cost sharing cases (single enterprise sharing, both enterprises sharing). Another obvious difference is that we proceed with research under uncertain demand and explore its effect on pricing and quality improvement decisions. Further, we also attempt to figure out whose cost sharing is more beneficial to the product’s quality and the supply chain members.

### 2.4 The research gaps and contributions

The existing literature regarding textile and garment supply chains mainly proceeded from an empirical perspective and rarely explored the operational decisions from the perspective of mathematical games. Our paper complements the existing literature by discussing the quality improvement and cost sharing behavior under uncertain demand with a game theory approach. We construct five game models to analyze the FM’s quality improvement and the GM’s and GR’s cost sharing behaviors, and then explore whose cost sharing is more conducive to the supply chain.

Most literature considering uncertain demand in supply chains focused on exploring its effect on the pricing decision and profits. Based on this literature, we further consider the impact of uncertain demand on quality improvement and cost sharing. Besides, this paper also considers the cost sharing behavior of different members to analyze whose cost sharing is more advantageous. A more detailed comparison is summarized in Table 1

**Table 1:** Comparison between the literature and our research.

Articles	Supply chain type	Research focus	Uncertain demand	Quality improvement	Cost sharing
[6]	garment SC	supplier selection			
[7]	textile and garment SC	supplier selection			
[13]	green SC	coordination problem	✔		
[14]	sustainable SC	extended warranty service	✔		
[22]	two-layer SC	supplier’s investment		✔	
[23]	e-commerce SC	information acquisition	✔		✔
[24]	dual-channel SC	competitive environment		✔	
[30]	low-carbon SC	cost sharing contract			✔
[32]	remanufacturing SC	process innovations			✔
Our paper	three-layer SC	cost sharing behaviors	✔	✔	✔

Note: SC = supply chain.

## 3 Problem description and demand function

**Fig 1:**
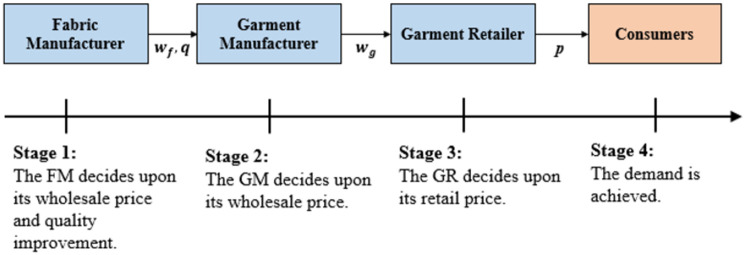
Supply chain structure and decision sequence. There are supply chain members and decision stages, and each member have own decision variable.

In this paper, we consider a textile and garment supply chain consisting of three members: an upstream FM, a midstream GM, and a downstream GR. The GM produces finished garments using fabric purchased from the FM and then sells them to end consumers through the GR. The three members engage in a Stackelberg game where the FM is the leader and the GM and GR are the followers. In this supply chain, the members are aware of perfect demand information and cooperate based on this information. More specifically, the supply chain structure and the event sequence are shown in Fig 1. The notations used in this work are summarized in Table 2.

**Table 2:** Notations used in this work.

Notations	Description
*a*	Market base
*θ*	The elasticity of price with respect to the demand
λ	The elasticity of quality improvement with respect to the demand
*δ*	The probability of high demand state, 0 < *δ* < 1
*η*	The proportion of cost shared by the GM, 0 < *η* < 1
*ϕ*	The proportion of cost shared by the GR, 0 < *ϕ* < 1
*c*_*f*_	The FM’s unit production cost
*c*_*g*_	The GM’s unit production cost
*μ*	The FM’s cost coefficient of quality improvement
Π_*FM*_	The FM’s profit
Π_*GM*_	The GM’s profit
Π_*GR*_	The GR’s profit
Decision Variables	
*w*_*f*_	The FM’s wholesale price
*w*_*g*_	The GM’s wholesale price
*p*	The GR’s retail price
*q*	The FM’s quality improvement

**Fig 2:**
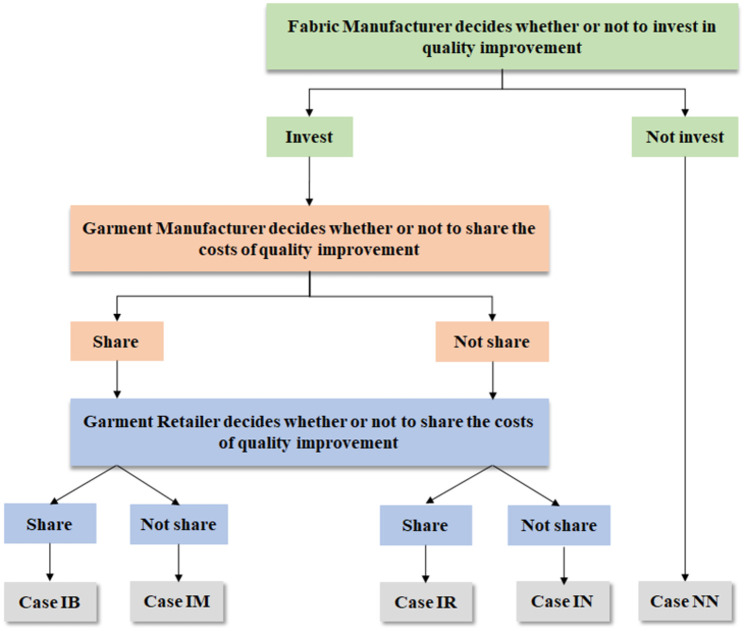
Five scenarios are considered in this supply chain. According to the three members’ different choices, it can be got the five cases we considered in this paper.

According to the FM’s choice of quality improvement and the GM’s and GR’s choices of cost sharing, five scenarios arise that can be studied, see Fig 2. In practice, there are many examples where GM is willing to share the cost of FM, while there are few cases where GR is willing to share the cost of FM. For example, Uniqlo, a clothing retail brand, recommends a “win-win” partnership with its suppliers, supporting suppliers in both software and hardware, such as sending “master teams” to the site to provide technical guidance and quality management at every stage from textile, dyeing, sewing, finishing to delivery. To transform the supplier’s factory, SHEIN will provide direct financial subsidies and on-site guidance to the supplier. For example, the supplier Qingmei’s factory used to print only about 2,000 meters of cloth every day, but after the transformation, the number has increased to 150,000 meters, and these changes cannot be achieved without SHEIN’s support.

Concretely, the five scenarios are as follows: (1) Case NN: the FM does not invest in quality improvement, and thus there is no cost sharing; (2) Case IN: the FM invests in quality improvement while neither the GM nor GR is willing to share the costs; (3) Case IR: the FM invests in quality improvement and only the GR is willing to share the costs; (4) Case IM: the FM invests in quality improvement and only the GM is willing to share the costs; (5) Case IB: the FM invests in quality improvement and both the GM and GR are willing to share the costs. The case that the GR shares the cost of quality improvement with the FM rarely happens, and we consider this situation in contrast to the GM’s cost sharing to explore a new cost sharing option and to seek the optimal quality improvement solution from the perspective of the whole supply chain. We want to theoretically determine the feasibility and advantages of GR sharing costs, to propose a new cost sharing approach to managers. Although the GR does not directly cooperate with the FM, the GR can also benefit from the FM’s quality improvement. Hence, the GR is willing to share the costs with the FM to incentivize its quality improvement. Under each case, we construct game models and derive the equilibrium solutions to discuss the impacts of uncertain demand on the pricing and quality improvement decisions as well as profits.

Referring to the previous literature [34, 38–40], we utilize price- and quality-sensitive demand:

$$\begin{array}{c} d=a-\theta p+\lambda q. \end{array}$$ (1)

Here, *θ* represents the price elasticity, and λ is the elasticity of quality improvements concerning the demand. *a* is the market base and is a random variable given by the high demand state *a*_*H*_ with probability *δ* and the low demand state *a*_*L*_ with probability 1 − *δ*, where *a*_*H*_ > *a*_*L*_ > 0. The assumption is similar to the previous literature [35, 41–43], and they also use the two demand states to simplify the reality situation and reveal that the assumption is sufficient to capture the impact of uncertain demand on decisions. For the same reason, we use the assumption of two demand states to simplify the models, and in practice, some enterprises will simply use high or low demand when forecasting demand.

## 4 Model analysis

### 4.1 No quality improvement (NN)

Under case NN, the FM does not invest in quality improvement, i.e., *μ* = 0, and thus there is no cost sharing. Based on the uncertain demand, we can get the expected profits of the three members:

$$\begin{array}{c} E\left(\Pi_{FM}\left(w_{f}\right)\right)=\left(E\left(a\right)-\theta p\right)(w_{f}-c_{f})=\left(\delta a_{H}+\left(1-\delta \right)a_{L}-\theta p\right)(w_{f}-c_{f}), \end{array}$$

$$\begin{array}{c} E\left(\Pi_{GM}\left(w_{g}\right)\right)=\left(E\left(a\right)-\theta p\right)(w_{g}-c_{g}-w_{f})=\left(\delta a_{H}+\left(1-\delta \right)a_{L}-\theta p\right)(w_{g}-c_{g}-w_{f}), \end{array}$$

$$\begin{array}{c} E\left(\Pi_{GR}\left(p\right)\right)=\left(E\left(a\right)-\theta p\right)(p-w_{g})=\left(\delta a_{H}+\left(1-\delta \right)a_{L}-\theta p\right)(p-w_{g}). \end{array}$$

The optimization problem can be depicted as follows:

$$\begin{array}{c} \max_{w_{f}}E\left(\Pi_{FM}\right)⟶\max_{w_{g}}E\left(\Pi_{GM}\right)⟶\max_{p}E\left(\Pi_{GR}\right). \end{array}$$

We can then obtain the equilibrium solutions using backward induction, as shown in Proposition 1.

**Proposition 1** *Under case NN, the optimal wholesale prices and the retail price are as follows:*

$$\begin{array}{c} w_{f}^{NN}=\frac{\delta a_{H}+\left(1-\delta \right)a_{L}+\theta (c_{f}-c_{g})}{2\theta },\;w_{g}^{NN}=\frac{3(\delta a_{H}+\left(1-\delta \right)a_{L})+\theta (c_{f}+c_{g})}{4\theta }, \end{array}$$

$$\begin{array}{c} p^{NN}=\frac{7(\delta a_{H}+\left(1-\delta \right)a_{L})+\theta (c_{f}+c_{g})}{8\theta }. \end{array}$$

Based on the above equilibrium solutions, we can derive the demand and the three members’ profits, as shown in Table 3.

**Table 3:** Equilibrium solutions under different cases.

	case *j* = *NN*	case *j* = *IN*	case *j* = *IR*
*d* ^*j*^	$\frac{\delta a_{H}+\left(1-\delta \right)a_{L}-\theta (c_{f}+c_{g})}{8}$	$\frac{\theta \mu (\delta a_{H}+\left(1-\delta \right)a_{L}-\theta \left(c_{f}+c_{g}\right))}{8\theta \mu -\lambda^{2}}$	$\frac{\theta \mu \left(1-\phi \right)(\delta a_{H}+\left(1-\delta \right)a_{L}-\theta (c_{f}+c_{g}))}{8\theta \mu \left(1-\phi \right)-\lambda^{2}}$
$\Pi_{FM}^{j}$	$\frac{(\delta a_{H}+\left(1-\delta \right)a_{L}-\theta {\left(c_{f}+c_{g})\right)}^{2}}{16\theta }$	$\frac{\mu (\delta a_{H}+\left(1-\delta \right)a_{L}-\theta {\left(c_{f}+c_{g})\right)}^{2}}{2(8\theta \mu -\lambda^{2})}$	$\frac{\mu \left(1-\phi \right)(\delta a_{H}+\left(1-\delta \right)a_{L}-\theta {\left(c_{f}+c_{g})\right)}^{2}}{2(8\theta \mu \left(1-\phi \right)-\lambda^{2})}$
$\Pi_{GM}^{j}$	$\frac{(\delta a_{H}+\left(1-\delta \right)a_{L}-\theta {\left(c_{f}+c_{g})\right)}^{2}}{32\theta }$	$\frac{2\theta \mu^{2}(\delta a_{H}+\left(1-\delta \right)a_{L}-\theta {\left(c_{f}+c_{g})\right)}^{2}}{{\left(8\theta \mu -\lambda^{2}\right)}^{2}}$	$\frac{2\theta \mu^{2}{\left(1-\phi \right)}^{2}(\delta a_{H}+\left(1-\delta \right)a_{L}-\theta {\left(c_{f}+c_{g})\right)}^{2}}{{\left(8\theta \mu \left(1-\phi \right)-\lambda^{2}\right)}^{2}}$
$\Pi_{GR}^{j}$	$\frac{(\delta a_{H}+\left(1-\delta \right)a_{L}-\theta {\left(c_{f}+c_{g})\right)}^{2}}{64\theta }$	$\frac{\theta \mu^{2}(\delta a_{H}+\left(1-\delta \right)a_{L}-\theta {\left(c_{f}+c_{g})\right)}^{2}}{{\left(8\theta \mu -\lambda^{2}\right)}^{2}}$	$\frac{\mu (2\theta \mu {\left(1-\phi \right)}^{2}-\lambda^{2}\phi )(\delta a_{H}+\left(1-\delta \right)a_{L}-\theta {\left(c_{f}+c_{g})\right)}^{2}}{2{\left(8\theta \mu \left(1-\phi \right)-\lambda^{2}\right)}^{2}}$

**Corollary 1** *The impacts of uncertain demand on the optimal decisions and profits are as follows:* $\frac{\partial w_{f}^{NN}}{\partial \delta }>0$ , $\frac{\partial w_{g}^{NN}}{\partial \delta }>0$, $\frac{\partial p^{NN}}{\partial \delta }>0$, $\frac{\partial d^{NN}}{\partial \delta }>0$, $\frac{\partial E(\Pi_{FM}^{NN})}{\partial \delta }>0$, $\frac{\partial E(\Pi_{GM}^{NN})}{\partial \delta }>0$, $\frac{\partial E(\Pi_{GR}^{NN})}{\partial \delta }>0$.

This corollary shows that as the probability of high demand state *δ* increases, the FM’s and GM’s wholesale prices both go up, and the GR’s retail price also increases. Moreover, the three members’ optimal profits all increase with the increase of *δ*. One possible reason is that an increase in *δ* represents that the market base *a* is more likely to be a high value *a*_*H*_. The raised wholesale prices and increased demand bring more profits for the three members.

### 4.2 Quality improvement without cost sharing (IN)

Under case IN, the FM invests in quality improvement, i.e., *μ* > 0, while neither the GM nor GR is willing to share the corresponding costs. The demand function is affected not only by the retail price but also by the quality improvement. Because the quality-improved product could attract more quality-sensitive consumers, making the increased demand. Based on the uncertain demand, we have the expected profits of the three members:

$$\begin{array}{c} E\left(\Pi_{FM}\left(w_{f},q\right)\right)=(w_{f}-c_{f})\left(E\left(a\right)-\theta p+\lambda q\right)-\frac{\mu q^{2}}{2}, \end{array}$$

$$\begin{array}{c} E\left(\Pi_{GM}\left(w_{g}\right)\right)=(w_{g}-c_{g}-w_{f})\left(E\left(a\right)-\theta p+\lambda q\right), \end{array}$$

$$\begin{array}{c} E\left(\Pi_{GR}\left(p\right)\right)=(p-w_{g})\left(E\left(a\right)-\theta p+\lambda q\right). \end{array}$$

The optimization problem can be depicted as follows: $\max_{w_{f},q}E\left(\Pi_{FM}\right)\to \max_{w_{g}}E\left(\Pi_{GM}\right)\to \max_{p}E\left(\Pi_{GR}\right)$.

Similarly, we can derive the equilibrium solutions using backward induction, see Proposition 2. Here, we assume that $\mu >\frac{\lambda^{2}}{8\theta }$ holds to ensure that the profit function is concave.

**Proposition 2** *Under case IN, the three members’ optimal decisions are as follows:*

$$\begin{array}{c} w_{f}^{IN}=\frac{4\mu (\delta a_{H}+\left(1-\delta \right)a_{L}-\theta c_{g})+c_{f}(4\theta \mu -\lambda^{2})}{8\theta \mu -\lambda^{2}},q^{IN}=\frac{\lambda (\delta a_{H}+\left(1-\delta \right)a_{L}-\theta (c_{f}+c_{g}))}{8\theta \mu -\lambda^{2}}, \end{array}$$

$$\begin{array}{c} w_{g}^{IN}=\frac{6\mu (\delta a_{H}+\left(1-\delta \right)a_{L})+(c_{f}+c_{g})(2\theta \mu -\lambda^{2})}{8\theta \mu -\lambda^{2}},p^{IN}=\frac{7\mu (\delta a_{H}+\left(1-\delta \right)a_{L})+(c_{f}+c_{g})(\theta \mu -\lambda^{2})}{8\theta \mu -\lambda^{2}}. \end{array}$$

Based on the above equilibrium solutions, we can derive the optimal demand and the three members’ profits, as shown in Table 3.

**Corollary 2** *(1) The impacts of uncertain demand on the optimal decisions and profits are as follows:* $\frac{\partial w_{f}^{IN}}{\partial \delta }>0$ , $\frac{\partial w_{g}^{IN}}{\partial \delta }>0$, $\frac{\partial q^{IN}}{\partial \delta }>0$, $\frac{\partial p^{IN}}{\partial \delta }>0$, $\frac{\partial d^{IN}}{\partial \delta }>0$, $\frac{\partial E(\Pi_{FM}^{IN})}{\partial \delta }>0$, $\frac{\partial E(\Pi_{GM}^{IN})}{\partial \delta }>0$, $\frac{\partial E(\Pi_{GR}^{IN})}{\partial \delta }>0$.

*(2) The impacts of quality improvement on the optimal decisions and profits are as follows:* $\frac{\partial w_{f}^{IN}}{\partial \mu }<0$ , $\frac{\partial w_{g}^{IN}}{\partial \mu }<0$, $\frac{\partial q^{IN}}{\partial \mu }<0$, $\frac{\partial p^{IN}}{\partial \mu }<0$, $\frac{\partial d^{IN}}{\partial \mu }<0$, $\frac{\partial E(\Pi_{FM}^{IN})}{\partial \mu }<0$, $\frac{\partial E(\Pi_{GM}^{IN})}{\partial \mu }<0$, $\frac{\partial E(\Pi_{GR}^{IN})}{\partial \mu }<0$.

Part (1) in this corollary shows that the probability of high demand state *δ* has a positive effect on the prices, quality, and profits, which is similar to those of Corollary 1. Part (2) reveals that, as the cost coefficient *μ* increases, the FM’s and GM’s wholesale prices, the quality improvement, and the GR’s retail price all decrease. That means that the cost coefficient *μ* has a negative effect on the three members’ profits. The higher the *μ*, the higher the cost of quality improvement. It can be easy to understand that the FM’s profit declines when the cost of quality improvement goes up. However, the GM’s profit declines due to the decreased wholesale price and the increased demand; the GR’s profit is descending because both the retail price and the demand decline.

### 4.3 Quality improvement with GR sharing the costs (IR)

Under case IR, the FM invests in quality improvement while only the GR is willing to share the costs. The demand function remains unchanged with that of case IN. Based on this, we get the three members’ expected profits:

$$\begin{array}{c} E\left(\Pi_{FM}\left(w_{f},q\right)\right)=(w_{f}-c_{f})\left(E\left(a\right)-\theta p+\lambda q\right)-\frac{1}{2}\left(1-\phi \right)\mu q^{2}, \end{array}$$

$$\begin{array}{c} E\left(\Pi_{GM}\left(w_{g}\right)\right)=(w_{g}-c_{g}-w_{f})\left(E\left(a\right)-\theta p+\lambda q\right), \end{array}$$

$$\begin{array}{c} E\left(\Pi_{GR}\left(p\right)\right)=(p-w_{g})\left(E\left(a\right)-\theta p+\lambda q\right)-\frac{1}{2}\phi \mu q^{2}. \end{array}$$

The equilibrium solutions are presented in Proposition 3. Here, the assumption $\mu >\frac{\lambda^{2}}{8(1-\phi )\theta }$ holds to ensure that the profit function is concave.

**Proposition 3** *Under case IR, the optimal wholesale prices, the quality improvement, and the retail price are as follows:*

$$\begin{array}{c} w_{f}^{IR}=\frac{4\mu \left(1-\phi \right)(\delta a_{H}+\left(1-\delta \right)a_{L}-\theta c_{g})+c_{f}(4\theta \mu \left(1-\phi \right)-\lambda^{2})}{8\theta \mu \left(1-\phi \right)-\lambda^{2}}, \end{array}$$

$$\begin{array}{c} q^{IR}=\frac{\lambda (\delta a_{H}+\left(1-\delta \right)a_{L}-\theta c_{f}-\theta c_{g})}{8\theta \mu \left(1-\phi \right)-\lambda^{2}}, \end{array}$$

$$\begin{array}{c} w_{g}^{IR}=\frac{6\mu \left(1-\phi \right)(\delta a_{H}+\left(1-\delta \right)a_{L})+(c_{f}+c_{g})(2\theta \mu \left(1-\phi \right)-\lambda^{2})}{8\theta \mu \left(1-\phi \right)-\lambda^{2}}, \end{array}$$

$$\begin{array}{c} p^{IR}=\frac{7\mu \left(1-\phi \right)(\delta a_{H}+\left(1-\delta \right)a_{L})+(c_{f}+c_{g})(\theta \mu \left(1-\phi \right)-\lambda^{2})}{8\theta \mu \left(1-\phi \right)-\lambda^{2}}. \end{array}$$

Based on the above equilibrium solutions, we derive the demand and the three members’ profits, as shown in Table 3.

**Corollary 3**. *(1) The impacts of uncertain demand on the optimal decisions and profits are as follows:* $\frac{\partial w_{f}^{IR}}{\partial \delta }>0,\frac{\partial w_{g}^{IR}}{\partial \delta }>0,\frac{\partial q^{IR}}{\partial \delta }>0,\frac{\partial p^{IR}}{\partial \delta }>0,\frac{\partial d^{IR}}{\partial \delta }>0,\frac{\partial E(\Pi_{FM}^{IR})}{\partial \delta }>0,\frac{\partial E(\Pi_{GM}^{IR})}{\partial \delta }>0,\frac{\partial E(\Pi_{GR}^{IR})}{\partial \delta }>0$.

*(2) The impacts of quality improvement on the optimal decisions and profits are as follows:* $\frac{\partial w_{f}^{IR}}{\partial \mu }<0,\frac{\partial w_{g}^{IR}}{\partial \mu }<0,\frac{\partial q^{IR}}{\partial \mu }<0,\frac{\partial p^{IR}}{\partial \mu }<0,\frac{\partial d^{IR}}{\partial \mu }<0,\frac{\partial E(\Pi_{FM}^{IR})}{\partial \mu }<0,\frac{\partial E(\Pi_{GM}^{IR})}{\partial \mu }<0$ ; $\frac{\partial E(\Pi_{GR}^{IR})}{\partial \mu }<0$.

Part (1) shows that the probability of high demand state *δ* has a positive effect on the decisions and profits, which is similar to those of cases IN and NN. This means that the impact of uncertain demand is not altered by the FM’s quality improvement and the GR’s cost sharing. In contrast, the GR’s cost sharing alters the impact of *μ* on herself profit, see part (2). Under case IR, the cost coefficient *μ* also plays a negative effect on the wholesale and retail prices, the quality improvement, and the three members’ profits.

**Corollary 4** *The impacts of cost sharing on the optimal decisions and profits are as follows:* $\frac{\partial w_{f}^{IR}}{\partial \phi }>0,\frac{\partial w_{g}^{IR}}{\partial \phi }>0,\frac{\partial q^{IR}}{\partial \phi }>0,\frac{\partial p^{IR}}{\partial \phi }>0,\frac{\partial d^{IR}}{\partial \phi }>0,\frac{\partial E(\Pi_{FM}^{IR})}{\partial \phi }>0,\frac{\partial E(\Pi_{GM}^{IR})}{\partial \phi }>0$ , $\frac{\partial E(\Pi_{GR}^{IR})}{\partial \phi }<0$.

This corollary indicates that the GR’s proportion of cost sharing *ϕ* has a positive effect on the wholesale and retail prices, quality improvement, and demand. The higher *ϕ*, the higher the quality improvement. This suggests the GR’s cost sharing is conducive to the FM’s quality improvement. In addition, the FM’s and GM’s profits are increasing with the proportion of cost sharing *ϕ*. However, the GR’s profit is negatively related to the proportion. Hence, the higher GR’s the proportion of cost sharing, the higher the FM’s and GM’s profits, and the lower the GR’s profit. Through the cost sharing behavior, the GR sacrifices her profits to bring more profits for the other members.

### 4.4 Quality improvement with GM sharing the costs (IM)

Under case IM, the FM invests in quality improvement, i.e., *μ* > 0, while only the GM is willing to share the costs. The demand function remains unchanged with that of case IN. Based on this, we have the expected profits of the three members:

$$\begin{array}{c} E\left(\Pi_{FM}\left(w_{f},q\right)\right)=(w_{f}-c_{f})\left(E\left(a\right)-\theta p+\lambda q\right)-\frac{1}{2}\left(1-\eta \right)\mu q^{2}, \end{array}$$

$$\begin{array}{c} E\left(\Pi_{GM}\left(w_{g}\right)\right)=(w_{g}-c_{g}-w_{f})\left(E\left(a\right)-\theta p+\lambda q\right)-\frac{1}{2}\eta \mu q^{2}, \end{array}$$

$$\begin{array}{c} E\left(\Pi_{GR}\left(p\right)\right)=(p-w_{g})\left(E\left(a\right)-\theta p+\lambda q\right). \end{array}$$

The equilibrium solutions are summarized in Proposition 4. Here, we assume that $\mu >\frac{\lambda^{2}}{8(1-\eta )\theta }$ holds to ensure that the profit function is concave.

**Proposition 4** *Under case IM, the optimal wholesale prices, the quality improvement, and the retail price are as follows:*

$$\begin{array}{c} w_{f}^{IM}=\frac{4\left(1-\eta \right)\mu (\delta a_{H}+\left(1-\delta \right)a_{L}-\theta c_{g})+c_{f}(4\left(1-\eta \right)\theta \mu -\lambda^{2})}{8\left(1-\eta \right)\theta \mu -\lambda^{2}}, \end{array}$$

$$\begin{array}{c} q^{IM}=\frac{\lambda (\delta a_{H}+\left(1-\delta \right)a_{L}-\theta c_{f}-\theta c_{g})}{8\left(1-\eta \right)\theta \mu -\lambda^{2}}, \end{array}$$

$$\begin{array}{c} w_{g}^{IM}=\frac{6\left(1-\eta \right)\mu (\delta a_{H}+\left(1-\delta \right)a_{L})+(c_{f}+c_{g})(2\left(1-\eta \right)\theta \mu -\lambda^{2})}{8\left(1-\eta \right)\theta \mu -\lambda^{2}}, \end{array}$$

$$\begin{array}{c} p^{IM}=\frac{7\left(1-\eta \right)\mu (\delta a_{H}+\left(1-\delta \right)a_{L})+(c_{f}+c_{g})(\left(1-\eta \right)\theta \mu -\lambda^{2})}{8\left(1-\eta \right)\theta \mu -\lambda^{2}}. \end{array}$$

According to the above equilibrium solutions, we derive the demand and the three members’ profits, as shown in Table 4.

**Table 4:** Equilibrium solutions under different cases.

	case *j* = *IM*	case *j* = *IB*
*d* ^*j*^	$\frac{\left(1-\eta \right)\theta \mu (\delta a_{H}+\left(1-\delta \right)a_{L}-\theta (c_{f}+c_{g}))}{8\left(1-\eta \right)\theta \mu -\lambda^{2}}$	$\frac{\theta \mu \left(1-\eta -\phi \right)(\delta a_{H}+\left(1-\delta \right)a_{L}-\theta (c_{f}+c_{g}))}{8\theta \mu \left(1-\eta -\phi \right)-\lambda^{2}}$
$\Pi_{FM}^{j}$	$\frac{\left(1-\eta \right)\mu (\delta a_{H}+\left(1-\delta \right)a_{L}-\theta {\left(c_{f}+c_{g})\right)}^{2}}{2(8\left(1-\eta \right)\theta \mu -\lambda^{2})}$	$\frac{\mu \left(1-\eta -\phi \right)(\delta a_{H}+\left(1-\delta \right)a_{L}-\theta {\left(c_{f}+c_{g})\right)}^{2}}{2(8\theta \mu \left(1-\eta -\phi \right)-\lambda^{2})}$
$\Pi_{GM}^{j}$	$\frac{\mu (4{\left(1-\eta \right)}^{2}\theta \mu -\eta \lambda^{2})(\delta a_{H}+\left(1-\delta \right)a_{L}-\theta {\left(c_{f}+c_{g})\right)}^{2}}{2{\left(8\left(1-\eta \right)\theta \mu -\lambda^{2}\right)}^{2}}$	$\frac{\mu (4\theta \mu {\left(1-\eta -\phi \right)}^{2}-\eta \lambda^{2})(\delta a_{H}+\left(1-\delta \right)a_{L}-\theta {\left(c_{f}+c_{g})\right)}^{2}}{2{\left(8\theta \mu \left(1-\eta -\phi \right)-\lambda^{2}\right)}^{2}}$
$\Pi_{GR}^{j}$	$\frac{{\left(1-\eta \right)}^{2}\theta \mu^{2}(\delta a_{H}+\left(1-\delta \right)a_{L}-\theta {\left(c_{f}+c_{g})\right)}^{2}}{{\left(8\left(1-\eta \right)\theta \mu -\lambda^{2}\right)}^{2}}$	$\frac{\mu (2\theta \mu {\left(1-\eta -\phi \right)}^{2}-\lambda^{2}\phi )(\delta a_{H}+\left(1-\delta \right)a_{L}-\theta {\left(c_{f}+c_{g})\right)}^{2}}{2{\left(8\theta \mu \left(1-\eta -\phi \right)-\lambda^{2}\right)}^{2}}$

**Corollary 5** *(1) The impacts of uncertain demand on the optimal decisions and profits are as follows:* $\frac{\partial w_{f}^{IM}}{\partial \delta }>0,\frac{\partial w_{g}^{IM}}{\partial \delta }>0,\frac{\partial q^{IM}}{\partial \delta }>0,\frac{\partial p^{IM}}{\partial \delta }>0,\frac{\partial d^{IM}}{\partial \delta }>0,\frac{\partial E(\Pi_{FM}^{IM})}{\partial \delta }>0,\frac{\partial E(\Pi_{GM}^{IM})}{\partial \delta }>0,\frac{\partial E(\Pi_{GR}^{IM})}{\partial \delta }>0$ .

*(2) The impacts of quality improvement on the optimal decisions and profits are as follows:* $\frac{\partial w_{f}^{IM}}{\partial \mu }<0,\frac{\partial w_{g}^{IM}}{\partial \mu }<0,\frac{\partial q^{IM}}{\partial \mu }<0,\frac{\partial p^{IM}}{\partial \mu }<0,\frac{\partial d^{IM}}{\partial \mu }<0,\frac{\partial E(\Pi_{FM}^{IM})}{\partial \mu }<0,\frac{\partial E(\Pi_{GR}^{IM})}{\partial \mu }<0$ ; when $0<\eta <\frac{1}{3}$, $\frac{\partial E(\Pi_{GM}^{IM})}{\partial \mu }>0$, when $\frac{1}{3}\leq \eta <1$, $\frac{\partial E(\Pi_{GM}^{IM})}{\partial \mu }<0$.

Part (1) reveals that the probability of high demand state *δ* has a positive effect on the decisions and profits, which is similar to those of cases IM, IN, and NN. This means that the impact of uncertain demand is not altered by the GM’s cost sharing. In contrast, the GM’s cost sharing alters the impact of *μ* on herself profit, see part (2). Under case IM, the cost coefficient *μ* also plays a negative effect on the wholesale and retail prices, the quality improvement, and the FM’s and GR’s profits. Differently, when the GM’s proportion of cost sharing $\eta <\frac{1}{3}$, the GM’s profit increases with respect to *μ*; otherwise, the GR’s profit decreases with respect to *μ*. Likely, the impact of cost coefficient *μ* on the GM’s profit is affected by her cost sharing behavior.

**Corollary 6** *The impacts of cost sharing on the optimal decisions and profits are as follows:* $\frac{\partial w_{f}^{IM}}{\partial \eta }>0,\frac{\partial w_{g}^{IM}}{\partial \eta }>0,\frac{\partial q^{IM}}{\partial \eta }>0,\frac{\partial p^{IM}}{\partial \eta }>0,\frac{\partial d^{IM}}{\partial \eta }>0,\frac{\partial E(\Pi_{FM}^{IM})}{\partial \eta }>0$ .

This corollary indicates that the GM’s proportion of cost sharing *η* has a positive effect on the wholesale and retail prices, quality improvement, and demand. The higher *ϕ*, the higher the quality improvement. This suggests that the GM’s cost sharing is also conducive to the FM’s quality improvement. Moreover, the FM’s profit is increasing with the proportion of cost sharing *η*.

### 4.5 Quality improvement with both GM and GR sharing the costs (IB)

Under case IB, the FM invests in quality improvement, and both the GM and GR are willing to share the costs. Similar to case IM, we can get the expected profits of the three members:

$$\begin{array}{c} E\left(\Pi_{FM}\left(w_{f},q\right)\right)=(w_{f}-c_{f})\left(E\left(a\right)-\theta p+\lambda q\right)-\frac{1}{2}\left(1-\eta -\phi \right)\mu q^{2}, \end{array}$$

$$\begin{array}{c} E\left(\Pi_{GM}\left(w_{g}\right)\right)=(w_{g}-c_{g}-w_{f})\left(E\left(a\right)-\theta p+\lambda q\right)-\frac{1}{2}\eta \mu q^{2}, \end{array}$$

$$\begin{array}{c} E\left(\Pi_{GR}\left(p\right)\right)=(p-w_{g})\left(E\left(a\right)-\theta p+\lambda q\right)-\frac{1}{2}\phi \mu q^{2}. \end{array}$$

We can then obtain the equilibrium solutions by maximizing the expected profits, as shown in Proposition 5. Similarly, we assume that $\mu >\frac{\lambda^{2}}{8\theta (1-\eta -\phi )}$ holds to ensure that the optimal solutions exist.

**Proposition 5** *Under case IB, the optimal wholesale prices, quality improvements, and the retail price are as follows:*

$$\begin{array}{c} w_{f}^{\text{IB}}=\frac{4\mu \left(1-\eta -\phi \right)(\delta a_{H}+\left(1-\delta \right)a_{L}+\theta c_{f}-\theta c_{g})-\lambda^{2}c_{f}}{8\theta \mu \left(1-\eta -\phi \right)-\lambda^{2}}, \end{array}$$

$$\begin{array}{c} q^{\text{IB}}=\frac{\lambda (\delta a_{H}+\left(1-\delta \right)a_{L}-\theta c_{f}-\theta c_{g})}{8\theta \mu \left(1-\eta -\phi \right)-\lambda^{2}}, \end{array}$$

$$\begin{array}{c} w_{g}^{\text{IB}}=\frac{2\mu \left(1-\eta -\phi \right)(3(\delta a_{H}+\left(1-\delta \right)a_{L})+\theta (c_{f}+c_{g}))-\lambda^{2}(c_{f}+c_{g})}{8\theta \mu \left(1-\eta -\phi \right)-\lambda^{2}}, \end{array}$$

$$\begin{array}{c} p^{\text{IB}}=\frac{\mu \left(1-\eta -\phi \right)(7(\delta a_{H}+\left(1-\delta \right)a_{L})+\theta (c_{f}+c_{g}))-\lambda^{2}(c_{f}+c_{g})}{8\theta \mu \left(1-\eta -\phi \right)-\lambda^{2}}. \end{array}$$

Based on the above equilibrium solutions, we derive the demand and the three members’ profits, as shown in Table 4.

## 5 Comparison analysis

In this section, we compare the equilibrium solutions obtained under five cases from the theoretical level, and the corresponding results are provided in propositions. For situations that are too complex to draw clear conclusions, we will analyze them through numerical experiments in the next section. The comparison analyses are conducted under the condition $\mu >max\{\frac{\lambda^{2}}{8(1-\phi )\theta },\frac{\lambda^{2}}{8(1-\eta )\theta }\}$.

**Proposition 6** *By comparing the equilibrium solutions obtained under cases NN, IN, IM, IR, and IB, we demonstrate that the following properties hold:*

*(1)* $w_{f}^{\text{IB}}>w_{f}^{\text{j}}>w_{f}^{IN}>w_{f}^{NN}$ , $w_{g}^{\text{IB}}>w_{g}^{\text{j}}>w_{g}^{IN}>w_{g}^{NN}$, *j* = *IM*, *IR*; If *η* > *ϕ*, then $w_{f}^{IM}>w_{f}^{IR}$ and $w_{g}^{IM}>w_{g}^{IR}$, otherwise, $w_{f}^{IM}\leq w_{f}^{IR}$ and $w_{g}^{IM}\leq w_{g}^{IR}$;
*(2)* *p*^IB^ > *p*^j^ > *p*^IN^ > *p*^NN^, *d*^IB^ > *d*^j^ > *d*^IN^ > *d*^NN^, *j* = *IM*, *IR*; If *η* > *ϕ*, then *p*^IM^ > *p*^IR^, otherwise, *p*^IM^ ≤ *p*^IR^;
*(3)* *q*^IB^ > *q*^j^ > *q*^IN^, *j* = *IM*, *IR*; If *η* > *ϕ*, then *q*^IM^ > *q*^IR^, otherwise, *q*^IM^ ≤ *q*^IR^.

Part (1) in Proposition 6 shows that the wholesale price *w*_*f*_ or *w*_*g*_ increases when the case shifts from NN to IN, IM (IR), and IB. That is to say, the wholesale price *w*_*f*_ or *w*_*g*_ is highest under case IB, while it is lowest under case NN. However, under which case of IM and IR, the wholesale price *w*_*f*_ or *w*_*g*_ is higher depending on the proportions of cost sharing *η* and *ϕ*. Concretely, when the GM’s cost sharing *η* is greater than the GR’s cost sharing *ϕ*, the two wholesale prices are higher under case IM; otherwise, the two wholesale prices are higher under case IR.

Part (2) indicates that the retail price is highest under case IB while it is lowest under case NN. This means that the FM’s quality improvement increases the retail price, and the GM’s or GR’s cost sharing also increases the retail price. When the FM invests in quality improvement, the costs are bound to increase. To cover the increased costs, the FM will raise the wholesale price; This, in turn, leads to a higher retail price to cope with a higher wholesale price. The GM’s or GR’s cost sharing will increase their costs and they need to set a higher wholesale price or retail price to cover the increased costs. The results also suggest that the demand goes up when the case shifts from NN to IN, IM (IR), and IB. In other words, when a product’s quality improves, more consumers are willing to purchase it. However, under which case of IM and IR, the retail price is higher depending on the proportion of cost sharing *η* and *ϕ*. To be specific, whose proportion of cost sharing is higher, and whose cost sharing leads to a higher retail price.

Part (3) reveals that the FM’s quality improvement is highest under case IB, while it is lowest under case IN. That is, no matter whether the GM or the GR shares the FM’s costs, the quality improvement is higher than that without cost sharing. Nevertheless, whose cost sharing is more beneficial to the quality depends on the proportion of cost sharing. When the GM’s cost sharing *η* is greater than the GR’s cost sharing *ϕ*, the GM’s cost sharing is more beneficial to the quality; otherwise, the GR’s cost sharing is more beneficial to the quality. In other words, the higher the proportion of cost sharing, the more benefits to the quality. In short, the cost sharing of supply chain members is beneficial to quality improvement.

**Proposition 7** *By comparing the FM’s expected profit obtained under cases NN, IN, IR, IM, and IB, the following properties hold:* $E\left(\Pi_{FM}^{\text{IB}}\right)>E\left(\Pi_{FM}^{\text{j}}\right)>E\left(\Pi_{FM}^{IN}\right)>E\left(\Pi_{FM}^{NN}\right)$ , *j* = *IM*, *IR*; when *η* > *ϕ*, $E\left(\Pi_{FM}^{IM}\right)>E\left(\Pi_{FM}^{IR}\right)$, otherwise, $E\left(\Pi_{FM}^{IM}\right)\leq E\left(\Pi_{FM}^{IR}\right)$.

Proposition 7 shows that the FM’s profit increases when the case shifts from NN to IN, IM (IR), and IB. This means that, although the quality improvement increases the FM’s costs, it also brings more profit. Furthermore, no matter whether the GM or GR shares the costs, the FM’s profit is always higher than that without cost sharing; when both the GR and GM share the costs, the FM’s profit is highest. Therefore, other members’ cost sharing behaviors are beneficial to the FM. However, whose cost sharing is more favorable for the FM depends on the proportion of cost sharing. When the GM’s proportion is higher, the FM benefits more from the GM’s cost sharing; otherwise, the FM benefits more from the GR’s cost sharing. Likely, the FM prefers one member, with a high proportion of cost sharing, to share the costs.

**Proposition 8** *By comparing the GM’s expected profit obtained under cases NN, IN, IR, IM, and IB, we obtain:* $E\left(\Pi_{GM}^{IN}\right)>E\left(\Pi_{GM}^{NN}\right)$ ; when *η* = *ϕ*, $E\left(\Pi_{GM}^{IM}\right)<E\left(\Pi_{GM}^{IR}\right)$; when $\phi \in (\frac{64\eta \theta^{2}\mu^{2}-4\left(\eta +2\right)\theta \lambda^{2}\mu +\lambda^{4}}{8\theta \mu (8\eta \theta \mu -\lambda^{2})},+\infty )\cap \left(0,1\right)$, $E\left(\Pi_{GM}^{\text{IB}}\right)>E\left(\Pi_{GM}^{IR}\right)$.

Proposition 8 indicates that the GM’s profit is higher under case IN than under case NN, which means that the FM’s quality improvement is also advantageous for the GM. Interestingly, when the GM’s and GR’s proportions of cost sharing are equal, the GM receives higher profit from the GR’s cost sharing. Moreover, when the GR’s proportion of cost sharing *ϕ* is greater than a threshold, the GM gains more profit from case IB than from case IR.

**Proposition 9** *By comparing the GR’s expected profit obtained under cases NN, IN, IR, IM, and IB, we get:* $E\left(\Pi_{GR}^{IM}\right)>E\left(\Pi_{GR}^{IN}\right)>E\left(\Pi_{GR}^{NN}\right)$ ; when *η* = *ϕ*, $E\left(\Pi_{GR}^{IM}\right)>E\left(\Pi_{GR}^{IR}\right)$.

Proposition 9 reveals that the GR’s profit increases when the case shifts from NN to IN and IM. This suggests that the FM’s quality improvement is also beneficial to the GR and that the GM’s cost sharing also generates more profit for the GR. Interestingly, when the GM’s and GR’s proportions of cost sharing are equal, the GR receives higher profit from the GM’s cost sharing. This result is similar to that of Proposition 8, and that one member receives more profit when the other member shares the costs.

## 6 Numerical analysis

In this section, we employ numerical analysis to illustrate the results more intuitively. According to the related literature [14, 27, 34] and the constraint conditions of Section 4, the parameter settings are as follows: *a*_*H*_ = 500, *a*_*L*_ = 200, *δ* = 0.5, *θ* = 2, λ = 1, *c*_*f*_ = 10, *c*_*g*_ = 6, *μ* = 100, *η* = 0.1, and *ϕ* = 0.2. We subsequently explore the impacts of key parameters *δ*, *μ*, *ϕ*, and *η* on the quality improvement and the three members’ expected profits.

### 6.1 Impacts of uncertain demand

**Fig 3:**
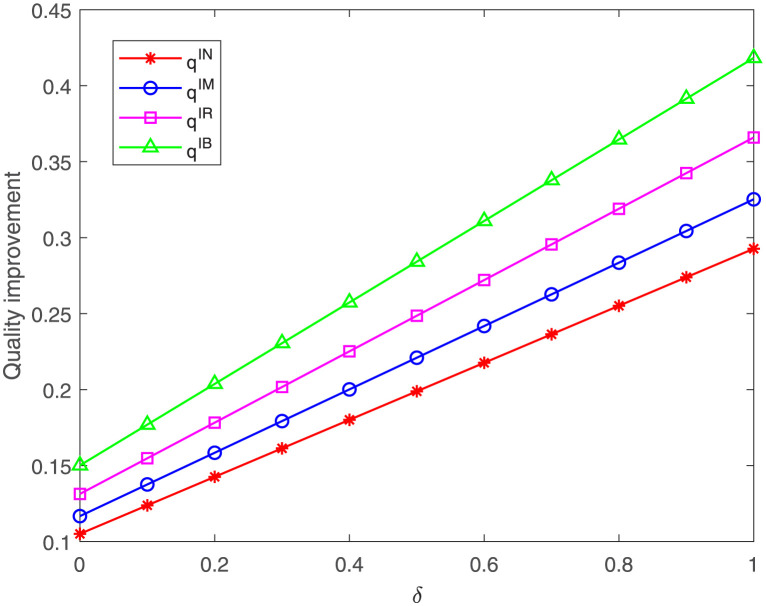
Impacts of *δ* on the quality improvement. Impact of *δ* on the quality improvement under cases IN, IM, IR, IB.

First, we explore how the probability of high demand status *δ* affects the quality improvement, as shown in Fig 3. It can be observed that, regardless of cases, the optimal quality improvement increases with the increase of *δ*. This means that, as the market demand increases, so will the quality improvement made by the FM. One possible reason is that when faced with a large market demand, manufacturers can gain greater benefits from quality improvement. When the demand for the improved product is relatively large, the FM is more likely to get a positive return from the quality improvement.

**Fig 4:**
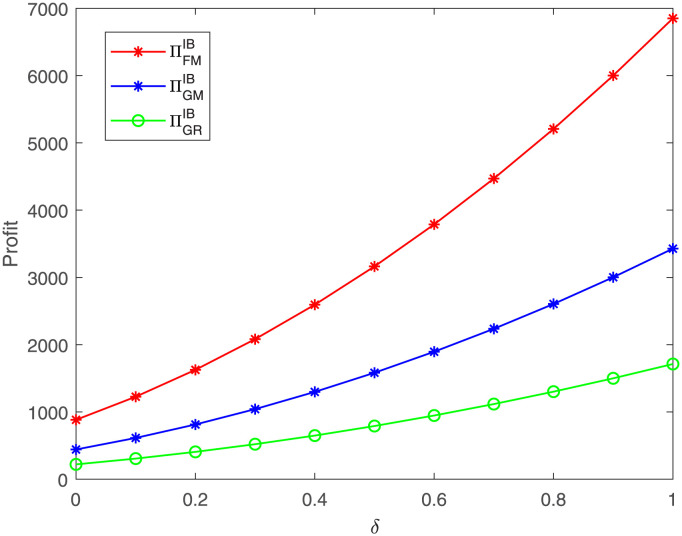
Impacts of *δ* on the members’ profits. The three members’ profit under case IB.

At the same time, we also explore the impact of *δ* on the three members’ profits, as shown in Fig 4. We conduct a series of numerical experiments under five cases and find the outcomes are similar, and thus we here only present the graph of case IB. The result indicates that the three members’ profits all go up as the probability of high demand status *δ* increases, which is consistent with the theoretical results in Section 4.

### 6.2 Impacts of cost coefficient *μ*

**Fig 5:**
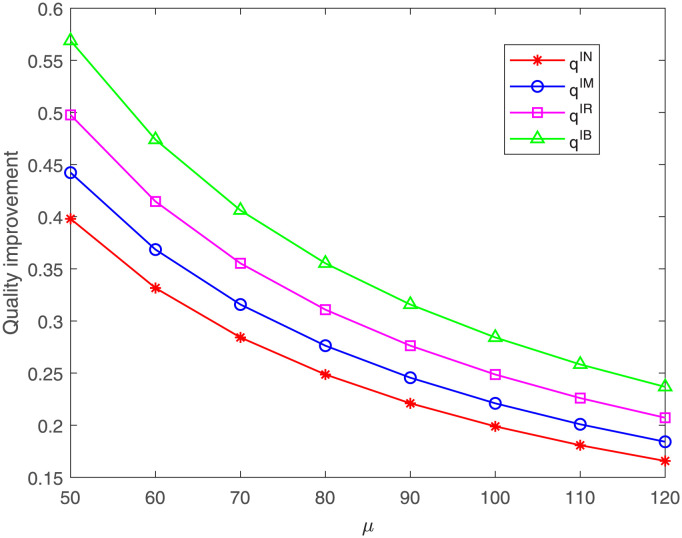
Impact of *μ* on the quality improvement. The quality improvement under cases IN, IM, IR, IB.

Here, we explore the impacts of cost coefficient *μ* on the quality improvement and the members’ profits. Fig 5 shows that, no matter under which case, the quality improvement is always descending with the increasing of cost coefficient *μ*, which is consistent with those corollaries in Section 4. Besides, we can find the quality improvement under case IB is always the highest and under case IN is always the lowest. It can observed that the quality improvement under case IR is higher than that under case IM, and this is because the parameter setting *ϕ* = 0.2 > *η* = 0.1, which is consistent with Proposition 6 in Section 5.

**Fig 6:**
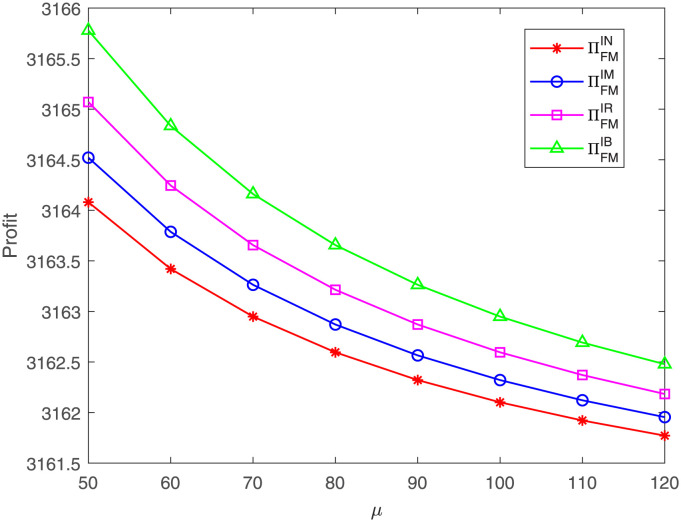
Impact of *μ* on the FM’s profit. The FM’s profit with respect to *μ* under cases IN, IM, IR, IB.

Fig 6 indicates that, regardless of which case, the FM’s profit decreases with the cost coefficient *μ*. No matter whether the GM or GR is willing to share the costs, the cost coefficient *μ* always hurts the FM’s profit, which is in line with those corollaries in Section 4. Similarly, the FM’s profit under case IB is always the highest and under case IN is always the lowest. Then the result presents the FM’s profit under case IR is higher than that under case IM, which is also the resulted by the parameter setting *ϕ* = 0.2 > *η* = 0.1. The outcomes conform to the Proposition 7 in Section 5.

**Fig 7:**
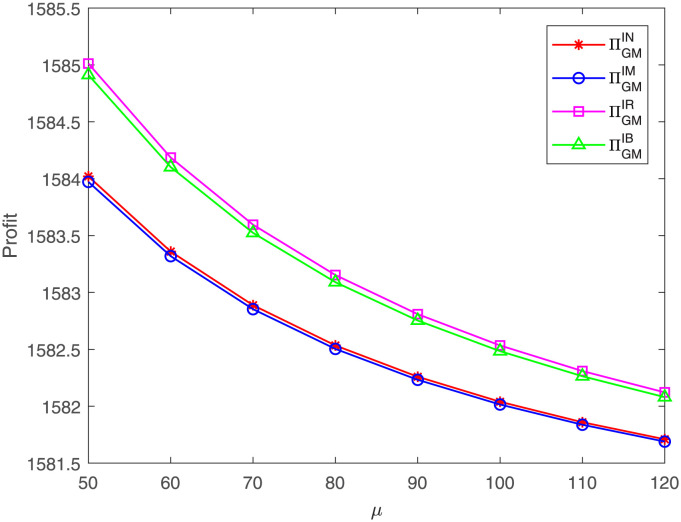
Impact of *μ* on the GM’s profit. The GM’s profit with respect to *μ* under cases IN, IM, IR, IB.

Fig 7 reveals that, regardless of which case, the GM’s profit decreases with the increase of the cost coefficient *μ*. No matter whether the GM or GR is willing to share the costs, the FM’s cost coefficient *μ* always has an indirect and negative effect on the GM’s profit, which is in line with those corollaries in Section 4. An interesting finding is that the GM’s profit under case IR is highest and under case IM is lowest under these parameter settings. That means, compared with no cost sharing, only the GR’s cost sharing or the both members’ cost sharing generates more profit for the GM, while GM’s cost sharing results in lower profit. This is possible because the GM’s cost sharing reduces her profits, but when the GR shares the cost, the GM does not bear the costs and can benefit through free riding.

**Fig 8:**
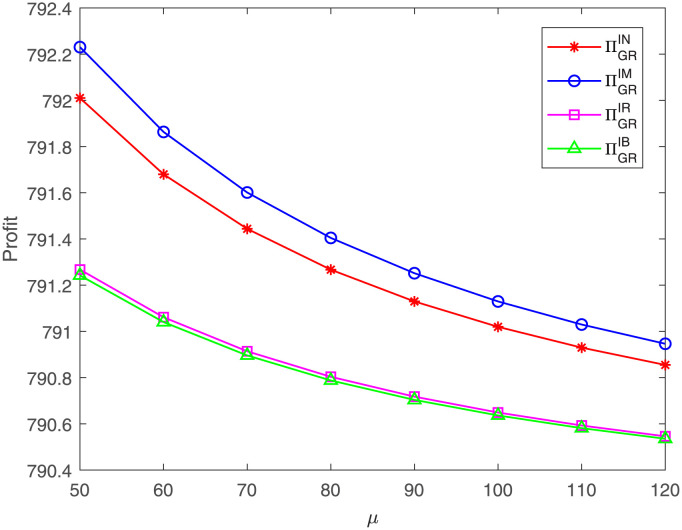
Impact of *μ* on the GR’s profit. The GR’s profit with respect to *μ* under cases IN, IM, IR, IB.

Fig 8 reflects that, regardless of which case, the GR’s profit decreases as the cost coefficient *μ* increases, which is in line with those corollaries in Section 4. This may be because the FM’s profit has fallen, leading to lower profits across the overall supply chain and lower profits in garment retailing. Furthermore, the GR’s profit under case IM is highest and under case IB is lowest under these parameter settings. That means, compared with no cost sharing, only the GM’s cost sharing generates more profit for the GR, while the two members’ cost sharing results in lower profit. Likely, when the GM shares the cost, the GR does not bear the costs and can benefit through free riding. In a word, the impacts of the cost coefficient *μ* on the FM, GM, and GR are similar.

### 6.3 Impacts of the proportion *η*

**Fig 9:**
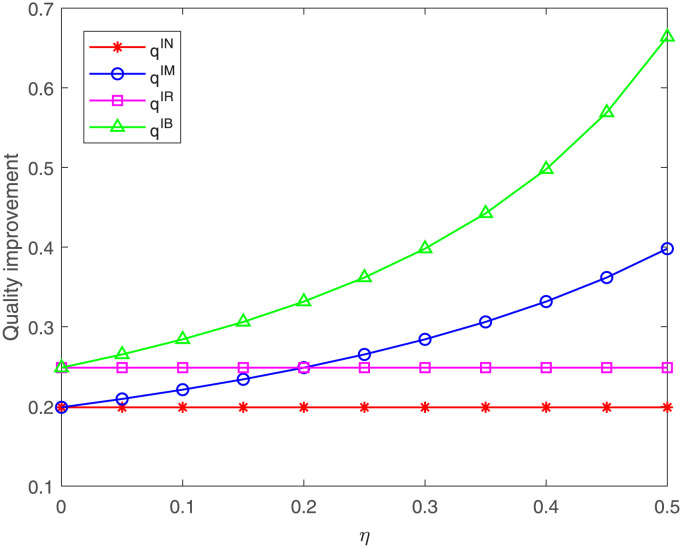
Impact of *η* on the quality improvement. The quality improvement with respect to *η* under cases IN, IM, IR, IB.

Next, we analyze the impacts of the proportion *η* on the quality improvement and the three members’ profits. From Fig 9, it can be found that no matter under case IM or IB, the quality improvement always increases with the increasing of the proportion *η*, which is consistent with Corollary 6 in Section 4. Besides, we can find the quality improvement under case IB is always higher than that under case IM. When the proportion *η* increases to 0.3 from 0, the quality improvement under case IM first is less than that under case IN, and then is higher than that under case IN but is less than that under case IR, and then the quality improvement under case IM is higher than that under case IR. That is to say, the quality improvement under which case is better hinging on the proportion *η*. However, the quality improvement under case IB is always the highest. These results are consistent with part (3) of Proposition 6 in Section 5.

**Fig 10:**
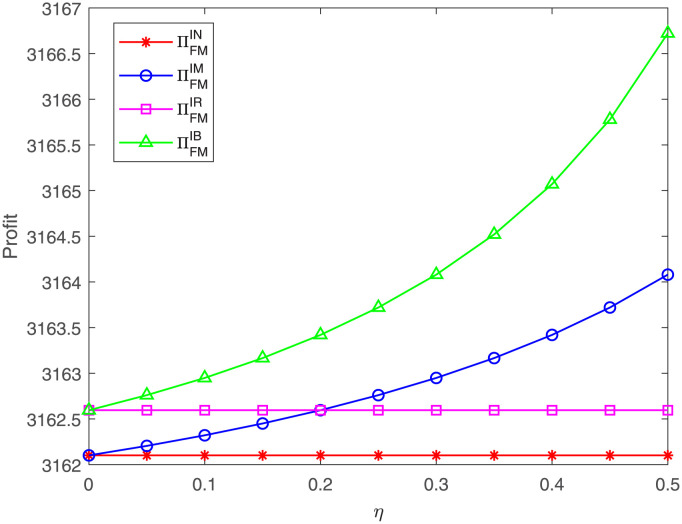
Impact of *η* on the FM’s profit. The FM’s profit with respect to *η* under cases IN, IM, IR, IB.

The FM’s profit concerning *η* under the different cases is presented in Fig 10. The results reveal that no matter under case IM or IB, the FM’s profit increases as the proportion *η* goes up, which is in line with Corollary 6 in Section 4. Moreover, the FM’s profit under case IB is always highest and under case IN is lowest. When the proportion *η* < *ϕ* = 0.2, the FM’s profit under case IR is less than that under case IM; when the proportion *η* increases to greater than 0.2, the FM receives a higher profit under case IM. This result is consistent with Proposition 7 in Section 5. The increase of *η* means that the costs shared by the GM are high and that the FM bears fewer costs which, in turn, adds to the FM’s profit. Therefore, the GM’s cost sharing is beneficial to the upstream FM.

**Fig 11:**
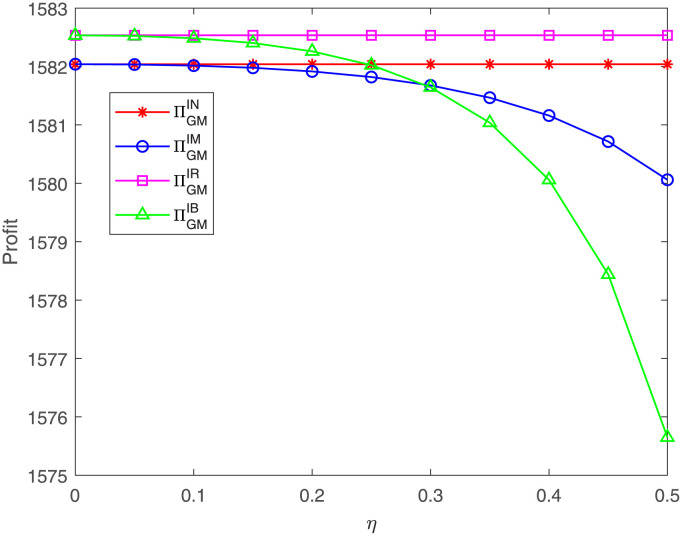
Impact of *η* on the GM’s profit. The GM’s profit with respect to *η* under cases IN, IM, IR, IB.

Fig 11 shows that the GM’s profit under case IM or IB is decreasing as the proportion *η* goes up, which is in line with Corollary 6 in Section 4. By comparing, we can find that the GM’s profit under case IR is the highest. When the proportion *η* is less than a threshold, the GM’s profit is lowest under case IM; when the proportion *η* is greater than the threshold, the GM’s profit is lowest under case IB. This result is consistent with Proposition 8 in Section 5. This means that the GM’s cost sharing behavior harms itself, and the more that the GM shares the costs, the less profit it gains. Moreover, the results remain unchanged even if the GR also shares the FM’s costs.

**Fig 12:**
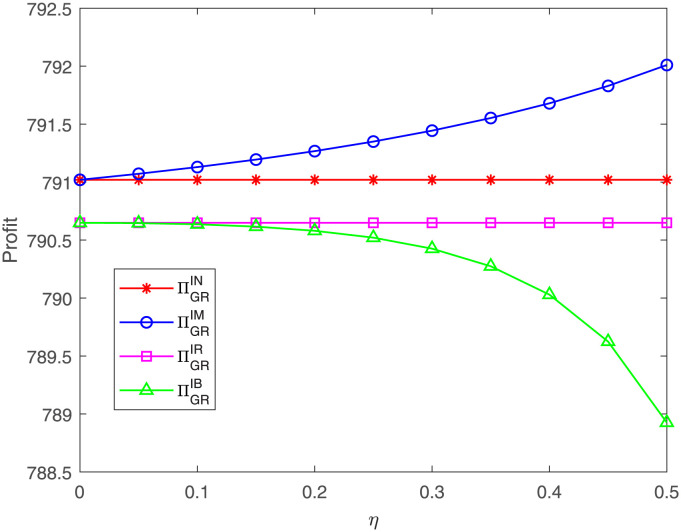
Impact of *η* on the GR’s profit. The GR’s profit with respect to *η* under cases IN, IM, IR, IB.

The GR’s profit concerning *η* under different cases is presented in Fig 12. The graph indicates that, as the proportion *η* goes up, the GR’s profit is increasing under case IM while is decreasing under case IB. The GR’s profit under case IM is highest while under case IB is lowest. These suggest that, when the GR does not take part in cost sharing, the GM’s cost sharing produces a positive effect on the downstream GR. However, when the GR also engages in cost sharing, the GM’s cost sharing has a negative effect on the downstream GR. These results are markedly different from the previous.

### 6.4 Impacts of the proportion *ϕ*

**Fig 13:**
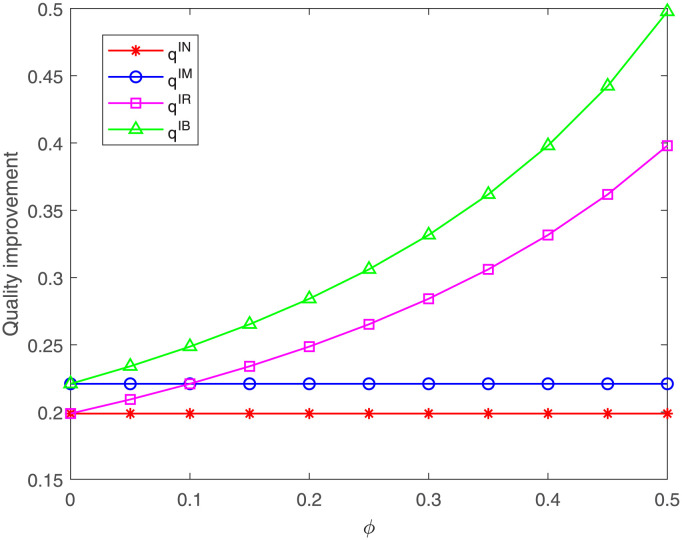
Impact of *ϕ* on the quality improvement. The quality improvement with respect to *ϕ* under cases IN, IM, IR, IB.

Then, we explore the impacts of the proportion *ϕ* on the quality improvement and the three members’ profits. From Fig 13, it can be found that no matter under case IR or IB, the quality improvement always increases with the increasing of the proportion *ϕ*, which is consistent with Corollary 4 in Section 4. Besides, we find the quality improvement under case IB is always the highest. When the proportion *ϕ* is less than *η* = 0.1, the quality improvement under case IR is less than that under case IM and is higher than that under case IN; When the proportion *ϕ* exceeds 0.1, the quality improvement under case IR is higher than that under case IM while is still less than that under case IB. That means the quality improvement under which case is better is related to the proportions *η* and *ϕ*. These results are consistent with part (3) of Proposition 6.

**Fig 14:**
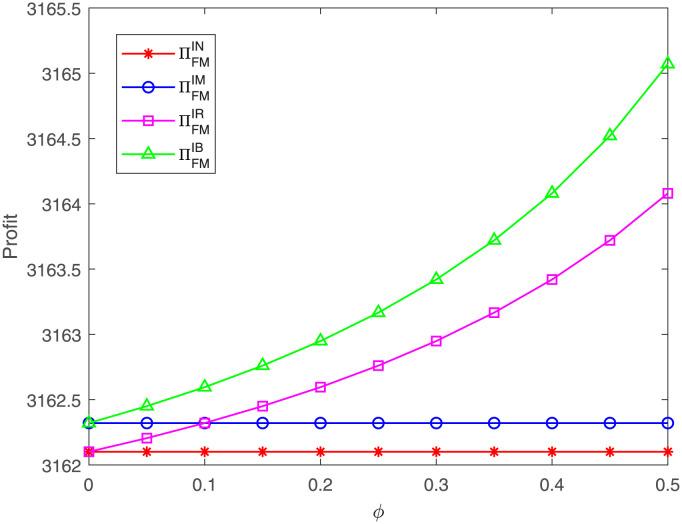
Impact of *ϕ* on the FM’s profit. The FM’s profit with respect to *ϕ* under cases IN, IM, IR, IB.

Similarly, from Fig 14, we observe that no matter under case IR or IB, the FM’s profit always increases with the increasing of the proportion *ϕ*, which is consistent with Corollary 4 in Section 4. Besides, we find that the FM’s profit under case IB is always the highest. When the proportion *ϕ* is less than *η* = 0.1, the FM’s profit under case IR is less than that under case IM and is higher than that under case IN; When the proportion *ϕ* exceeds 0.1, the FM’s profit under case IR is higher than that under case IM while is still less than that under case IB. That means, from which case of IM and IR, the FM receives higher profit is related to the proportions *η* and *ϕ*. These results are consistent with Proposition 8 in Section 5.

**Fig 15:**
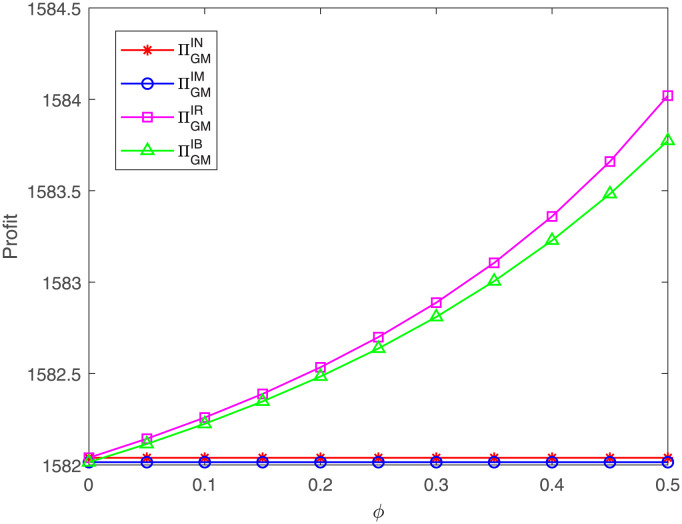
Impact of *ϕ* on the GM’s profit. The GM’s profit with respect to *ϕ* under cases IN, IM, IR, IB.

Fig 15 shows that the GM’s profit under case IR or IB is increasing as the proportion *ϕ* goes up, which is in line with Corollary 6 in Section 4. By comparing, we can find that the GM’s profit under case IR is the highest. This result is consistent with Proposition 8 in Section 5. This means that the GR’s single cost sharing benefits the GM, and the more that the GR shares the costs, the more profit the GM gains. Moreover, the results remain unchanged even if the GM also shares the FM’s costs.

**Fig 16:**
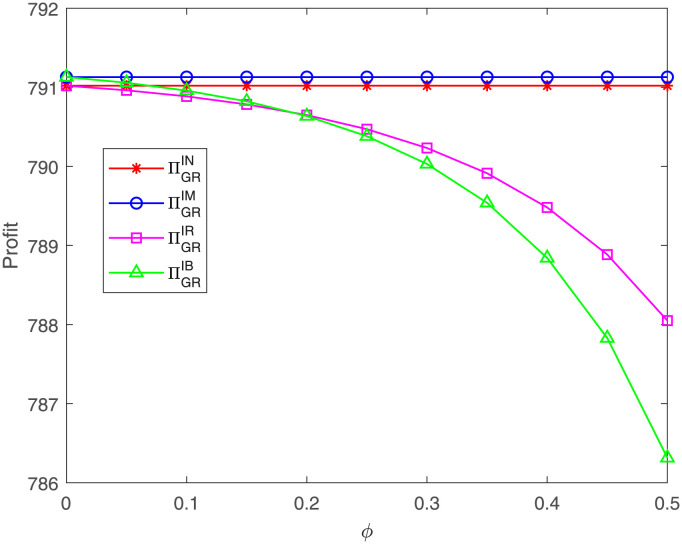
Impact of *ϕ* on the GR’s profit. The GR’s profit with respect to *ϕ* under cases IN, IM, IR, IB.

The GR’s profit concerning *η* under different cases is presented in Fig 16. The graph indicates that, as the proportion *ϕ* goes up, the GR’s profit decreases under case IR and IB. Moreover, the GR’s profit under case IM is the highest. When the proportion *ϕ* is less than a threshold, the GR’s profit under case IR is less than that under case IB. When the proportion *ϕ* exceeds the threshold, the GR’s profit under case IR is higher than that under case IB. That means, when the proportion *ϕ* is relatively small, both members’ cost sharing could bring more profit for the GR; if the proportion *ϕ* is relatively great, the GR’s single cost sharing could bring more profit for herself. But the profit is still less than that under case IM.

## 7 Conclusions

In this paper, we consider a textile and garment supply chain with three members: an upstream FM, a midstream GM, and a downstream GR. First, we construct five game models (i.e., NN, IN, IR, IM, IB) considering different investment choices and different cost sharing cases and then derive the optimal pricing and quality decisions. Second, we examine the optimal quality improvement decisions and the expected profits under uncertain demand, aiming to provide some management advice for enterprises. Third, we compare and analyze the impacts of different members’ cost sharing on the quality and the profits, and obtain some conditions under which each member could gain more profit, and thus guide enterprises in practice to make decisions.

### 7.1 Findings

First, regardless of which case, as the probability of high demand status increases, the quality improvement and the three members’ expected profits all go up. The FM’s cost coefficient of quality improvement has a negative effect on the quality improvement and the supply chain members’ profits. Moreover, the GM’s proportion of cost sharing has a positive effect on the quality improvement and the FM’s profit while hurts the GM’s profit. Differently, under case IM, the GM’s proportion of cost sharing has a positive effect on the GR’s profit while has a negative effect under case IB. In addition, the GR’s proportion of cost sharing has a positive effect on the quality improvement and the FM’s and GM’s profits while hurts the GR’s profit.

Second, the demand goes up when the case shifts from NN to IN, IM (IR), and IB. This means that quality improvement could attract more quality-sensitive consumers. It can be found that the FM’s quality improvement is highest under case IB and is lowest under case IN. As for which case of IM and IR, the quality improvement is higher depending on the two members’ proportions. A higher proportion leads to a higher quality improvement.

Third, the FM’s profit increases when the case shifts from NN to IN, IM (IR), and IB. This means that quality improvement and cost sharing create more profit for the FM, and those who choose to improve the quality can benefit. Whose cost sharing is more profitable for the FM depends on the GM’s and GR’s proportions of cost sharing. When the GM’s proportion is higher, the FM benefits more from the GM’s cost sharing; otherwise, the FM benefits more from the GR’s cost sharing. But, in general, both members’ cost sharing performs better than one member’s cost sharing in raising the FM’s profit.

Finally, the GM’s profit under case IN is always higher than that under case NN, and that is the GM benefits more from the upstream FM’s quality improvement. However, whose cost sharing can create more profit for the GM is uncertain and depends on the proportions of cost sharing. Numerical analysis shows that, the GM’s profit under case IR is always highest, and when the GM’s proportion of cost sharing is relatively low, both members’ cost sharing performs better than only the GM’s cost sharing in raising the latter’s profit. In addition, the GR’s profit increases when the case shifts from NN to IN, and IM, which means the upstream FM’s quality improvement also brings more profit for the GR. Compared with no cost sharing, the GM’s cost sharing benefits the GR. Besides, we find that the GR’s profit under case IM is always highest, and when the GR’s proportion of cost sharing is relatively low, both members’ cost sharing performs better than only the GR’s cost sharing in raising the GR’s profit.

### 7.2 Managerial implications

According to the above research findings, we can get some management insights, which can provide theoretical reference for the pricing and quality decisions of enterprises in practice, and guide the quality improvement of textile and garment supply chain enterprises and the cost sharing choice among members. From the perspective of product quality, the FM should invest in improving the quality, which not only benefits himself but also benefits the other members. On the other hand, the GM’s or GR’s cost sharing is conducive to quality improvement compared with no cost sharing, but the benefit is weaker than that under both members’ cost sharing. Hence, to get a higher quality, the FM should encourage the other members to share the cost. Not only the GM working directly with the FM, but also the downstream GR can be encouraged to share the cost.

From the perspective of the profits, the FM should choose quality improvement, which could benefit all the supply chain members compared with no investment. That is to say, the supply chain members are better off under the upstream FM’s investment. Without cost sharing, the GM and GR are free-riding because they benefit from the FM’s quality improvement at no cost. Thus, if the GM and GR do not share the FM’s costs, it could discourage the latter’s motivation for quality improvement. The GM and GR should encourage and support the FM to improve product quality and to get a win-win situation. For example, clothing brands such as UNIQLO and Zara have many upstream suppliers offering diversified fabrics. In order to obtain higher-quality fabrics, they can encourage upstream suppliers to improve the quality of raw materials by sharing research and development costs.

One incentive is to share the FM’s investment costs, as for the two members how to choose cost sharing strategy depends on the proportions that they are willing to afford. Compared with both members’ cost sharing, the GM (GR) benefits most from the GR’s (GM’s) cost sharing. That suggests that when one member is sharing the costs, the other member is better without sharing the costs. If one member has shared the costs, whether the other member engaging in cost sharing could benefit the former depends on their proportions. Specifically, when the GM chooses to share the costs and the proportion is relatively low, the GR joining in cost sharing is beneficial to the former; otherwise, is harmful to the former. Likewise, when the GR chooses to share the costs and the proportion is relatively low, the GM joining in cost sharing is beneficial to the former; otherwise, is harmful.

### 7.3 Further research

Although our work has produced some meaningful results, there are still some limitations, and which provide further research directions. Specifically, this paper only considers the FM as a leader and the GM and GR as followers, ignoring other power structures. For example, in cases where one GM or GR is the leader and one FM is the follower. In addition, the research can be extended to more complex supply chain structures, such as that concerning competing GMs or GRs, we could then continue to discuss enterprises’ quality improvement decisions under uncertain demand.
